# GFAP and alpha1a-AR staining and nuclear morphometry of oligodendrogliomas by confocal microscopy and image analysis: useful parameters for predicting survival in oligodendrogliomas

**DOI:** 10.1186/1746-1596-3-S1-S26

**Published:** 2008-07-15

**Authors:** Ernesto Moro-Rodríguez, Javier Figols, Mariano Alvira, José A Uranga-Ocio, Eduardo García-Poblete

**Affiliations:** 1 Universidad Rey Juan Carlos, Faculty of Health Sciences. Av/Atenas s/n. E-28922 Madrid, Spain; 2 Hospital Universitario Marqués de Valdecilla, Santander, Spain; 3 Pathologist, LifeSpan BioSciences, Inc. Seattle, WA, USA

## Abstract

**Objective:**

This study attempts to evaluate the GFAP and alpha1a-AR staining and morphometrical nuclear features of oligodendrogliomas and their prognostic implications as compared to present histopathology classification and their survival outcome.

**Study design:**

Surgical specimens from 24 patients with oligodendrogliomas during the period 1981–2000 were included. These cases were classified into two groups defined by the grade of the neoplasm: Group I: oligodendrogliomas grade II; Group II: oligodendrogliomas grade III and two groups based on the outcome status: Group of the alive cases and group of the death cases. Death rate for the groups were obtained by patients' charts. Descriptive statistics were used to examine the groups with respect to the morphometrical nuclear variables; area, perimeter, aspect, axes (major and minor), diameters (max, mean and min.), radius (max. and min.) margination, ratio of perimeter-area, roundness and sizes (length and width). In addition, an immunofluorescence method for GFAP and 1a-AR were performed and their area, density and intensity of staining were analyzed.

**Results:**

Semiautomated quantitative morphometrical results showed that the variables of nuclear area (GII 48.87 μm2 vs. GIII 43.45 μm2 p-value = 0.02), aspect (GII 1.39 vs. GIII 1.55 p-value = 0.03), axis minor (GII 6.66 μm vs. GIII 6.01 μm p-value = 0.003), diameter minor (GII 5.93 μm vs. GIII 5.27 μm p-value = 0.002), radius minor (GII 2.64 μm vs. GIII 2.25 μm p-value = 0,003), perimeter-area (GII 0.0007 vs. GIII 0.0006 p-value = 0.04), size width (GII 6.60 μm vs. GIII 5.96 μm p-value = 0,003), and density of alpha1a-AR staining (GII 121.38 vs. GIII 146.03 p-value = 0.05) were statistically significant in regard of grade; and that the sum of density of GFAP (p-value = 0.01) and the intensity of alpha1a-AR (p-value = 0.01) were statistically significant in predicting survival.

**Conclusion:**

These results suggest that some nuclear morphometrical features and the GFAP and alpha1a-AR immunofluorescence staining may be useful parameters for predicting survival in oligodendrogliomas.

## Introduction

Oligodendrogliomas (OD) are brain tumours predominantly composed of neoplastic oligodendroglial cells. They occur mainly in patients 30–40 years old. They usually originate in the frontal lobe of the brain and these patients usually present with chronic focal symptoms and have a protracted clinical course. In other patients, OD grows very quickly resulting in a fatal outcome. The WHO classification grades OD into three groups: grades I and II represent 90% of all OD and grade III include mixed oligodendroastocitomas and the malignant forms of OD [1]. However, most ODs are tumours with unpredictable outcomes.

As previously has been reported by Nafe, the precise description of the planar shapes of tumour cell nuclei by light microscopy is of crucial relevance for the investigation of outcome in tumours because the pleomorphism of nuclear shapes must be considered for typing, grading and prognostic assessment of many tumour types [2].

Previous studies had shown a detailed nuclear shape analysis by means of Fourier analysis, and compared other size and nuclear shape parameters with the measurement of DNA index by DNA densitometry between different tumour grades in oligodendrogliomas [2-4]. In order to increase optimization of nuclear shape analysis in this neuro-oncology study, we used here a fluorescent and confocal methodology and a semiautomatic quantitative analysis to improve the data obtained. The advantage of these techniques is that it is possible to segment the nucleus of each image in a more efficient way.

In the present study we performed two pairwise comparisons and tested the discriminatory power of different nuclear shape characteristics for each of these comparisons: (1) Oligodendrogliomas WHO grade II vs. WHO grade III; (2) comparison between the outcome survival status (patients with oligodendrogliomas that survived after five years vs. patients that died before five years) in order to find significant prognostic features. In addition, we used two antibodies to mark oligodendroglial and astrocytic cells. A rabbit plyclonal antibody (379-LP1248) against a unique peptide sequence of alpha1a-adrenoceptor (alpha1a-AR) and the glial fibrillary acid protein (GFAP) were colocalized by confocal microscopy.

Student's paired and two tails t-test were used to compare the mean values of the parameters studied.

## Materials and methods

Twenty-four oligodendrogliomas+ and two relapses were retrieved from the files of the Department of Pathology at "Marques de Valdecilla Hospital", Santander (Spain), over a period of more than ten years, beginning in 1981. Formalin-fixed, paraffin-embedded tumour samples from our files were employed. The tumours were characterized according to age, sex, year of diagnosis and histological degree in accordance with the WHO classification and outcome. All tumours underwent routine histological examination, including fixation in buffered formalin (4%).

Immunofluorescence and immunohistochemical techniques were used to co-localize the expression of 379-LP1248 and GFAP (Ab1 – Glial Fibrillary Acidic Protein – Bionova MS-280-R7). Propidium iodide was used for counterstaining DNA. Antibody 379-LP1248, recognizing a 16 amino acid peptide located in the third cytoplasm loop of the alpha1a-AR, was purchased from Life Span Biosciences, Seattle (WA).

Titration of antibody 379-LP1248 without antigen retrieval was carried out. The best results were obtained at 1/750 – 1/1000 dilution. Against the 379-LP1854, a secondary antibody Alexa Fluor 488 goat anti rabbit IgG(H+L) conjugated (Molecular Probes – Lab Net A-11008) was used at 1/100 and Alexa Fluor 633 rabbit anti mouse IgG(H+L) conjugated (Molecular Probes – Lab Net A-21063) at 1/100. Propidium iodide was used for 5 minutes.

A light-microscopic immunohistochemical study in an Axioplan/Axiophot 2 (Zeiss) was assessed semi-quantitatively by strength of staining (0 no stain, 1 weak, 2 moderate, and 3 strong). In addition, a confocal immunofluorescence quantitative study was performed in a Laser Scanning Microscope LSM 510 META (Zeiss) with a laser line of argon (458/488/514 nm 25 mW) and two laser lines of helium-neon (543 nm 1 mW; 633 nm, 5 mW). For this second purpose, we captured images from each case with three different optical magnifications, objectives: ×20 plan-Apochromat numerical aperture 0.75; ×40 plan-Neofluor numerical aperture 1.3 oil and ×63 plan-Apochromat numerical aperture 1.4 oil. We got an average of 7 pictures per case and a total number of 167 images for our statistical analysis. Between these images we always carried out a tile scan of 3 × 3 or 4 × 4 with 20× or 40× according to each case. From the 167 images collected, we recorded the mean, standard deviation and sum of the fluorescent intensities, area and density of expression for each channel ignoring the intensities lower than 4%. Following this method, we got nine different numerical values related to the immunoreactivity for the two primary antibody studied (GFAP and alpha1a-AR), and for the propidium iodade.

Finally, nuclear area, perimeter, aspect, axes (major and minor), diameters (max, mean and min.), radius (max. and min.) margination, ratio of perimeter-area, roundness and sizes (length and width) were measured by means of system for automated image analysis (Image Pro Plus – Media Cybernetics) based on a single tiff picture (512 × 512 pixels) per case at magnification ×400. In each case around 150 and 300 nuclei from the most cellular area were measured.

The statistical analysis was carried out using R software for Linux. For the univariate statistical analysis, the t-test was performed (two-sample t). A level of significance of p < 0.05 was adopted.

## Results

In this study we have investigated 24 patients and 2 recurring cases with clinical follow-up between three and seven years respectively after the original diagnosis. The study included 12 women and 12 men with ages ranging from 17 to 64 years (mean 37,69 F: 39,66 and M: 35,67). The follow-up time of all the patients was over five years. Fifteen patients (62,5%) died (6 W and 9 M), including six patients with recurring neoplasm. Only one patient died before ten months after the diagnosis. Among the nine surviving patients (6 W and 3 M), seven were free of disease (5 W and 2 M). Histological grade according to the WHO classification included 14 cases with grade II and 10 cases with grade III.

The quantitative analysis showed that the mean value of the nuclear area was 44.46 μm2; the mean nuclear perimeter value was 24.12 μm; the mean aspect value was 1.457; the axes (major and minor) values were 9.040 μm and 6.415 μm respectively; the diameters (max, mean and min.) values were 9.040 μm; 7.208 μm and 6.237 μm respectively; the radius (max. and min.) values were 4.690 μm and 2.493 μm respectively; the margination value was 0.8610; the ratio of perimeter-area was 0.0007145; the roundness value was 1.084 and the length and width sizes values were 8.785 μm and 6.359 μm respectively.

No statistical differences were found when comparing the nuclear parameters between the patients with oligodendrogliomas that survived to the tumour vs. patients whom did not survive the tumour.

Antibody 379-LP1248 consistently stained interfascicular and satellite oligodendrocytes in all human and guinea pig samples studied. The staining was not limited to the cell cytoplasm but, rather, it extended to thin branching oligodendroglial processes involving the neuropil in gray matter areas and myelinated fibers in the white matter. Occasionally some fibrous astrocytes also appear to faintly stain with antibody 379-LP1248, as confirmed by confocal microscopy and colocalized with GFAP. The predominant stained cell population consisted of cells morphologically identified as oligodendroglial in origin and did not exhibit colocalization with GFAP.

Light microscopic immunohistochemistry showed for alpha1a-AR a moderate staining of most cells in 12 (8 grade II/4 grade III) out of 26 OD tumours and strong staining in 9 (6 grade II/3 grade III).

Unvaried inferential tests comparing the mean intensities of fluorescence under LSM gives a mean of 566.07 for 379-LP1248, 243.62 for GFAP and 537.92 for IP with a t-value of 24.150, 21.082 and 23.836 respectively. Pearson's correlation for bivariate relationships was significant at 0.01 level for alpha1a-AR and GFAP.

The quantitative measurements of the immunofluorescence found significant differences comparing the mean values of the groups of oligodendrogliomas grade II versus grade III by means of the alpha1a-AR density. In relation with the outcome survival status, the patients with oligodendrogliomas that survived to the tumour had a lower sum of GFAP density comparing with the no survival group (p < 0.05).

## Discussion

Previous studies have shown that a detailed nuclear shape analysis can detect morphological differences between different tumour grades of brain tumours. In this way, Fourier analysis provided an optimal statistical discrimination between data sets from proliferating and non-proliferating tumour cell nuclei [2]. Other authors already concluded that quantitative parameters concerning nuclear size and shape could help to discriminate between groups of patients with different survival periods even in low-grade tumours [3]. Cytometry analysis found that the mean nuclear area of patients with glioblastomas multiform, anaplastic gliomas and low-grade glioma was significantly larger in the SG2M phase cells than those on G0G1 phase cells [4]. In the present study we focused exclusively on nuclear shape parameters comparing oligodendrogliomas based on their grade. Using a confocal microscopy approach we have found that there are some nuclear variables as the area; aspect; axis, diameter and radius minor; perimeter-area, size and width that were statistically significantly related to the grade of the tumour. These findings are of great value to avoid interobserver variability.

Using an antibody sensitive to a unique peptide sequence of alpha1a-AR we have demonstrated immunolocalization of this receptor in satellite and interfascicular oligodendrocytes in normal CNS tissue samples and in neoplastic oligodendroglial cells in human oligodendrogliomas. Moreover, following a quantitative approach with confocal microscopy, we showed that grade III OD exhibited greater staining for alpha1a-AR receptors than grade II OD. The non-specific staining with propidium iodade, used as an internal control, validated our methodology.

In summary: 1) Semiquantitative morphometrical results showed that the variables of nuclear area, aspect, axis minor, diameter minor, radius minor, perimeter-area, and size width were statistically significant in regard of OD grade. 2) The patients with oligodendrogliomas that survived to the tumour had a lower sum of GFAP density comparing with the no survival group. 3) We have shown immunoreactivity to a novel antibody to alpha1a-AR in oligodendrocites in normal SNC and in oligodendrogliomas. In addition and thanks to an immunofluorescence quantitative approach, we have observed that grade III OD exhibit greater presence of alpha1a-AR than grade II OD.

These results suggest that certain nuclear morphometrical features and the GFAP and alpha1a-AR immunofluorescence may be useful parameters in defining the nuclear grade and survival outcome of OD.
